# Morning glory species co‐occurrence is associated with asymmetrically decreased and cascading reproductive isolation

**DOI:** 10.1002/evl3.205

**Published:** 2020-11-18

**Authors:** Kate L. Ostevik, Joanna L. Rifkin, Hanhan Xia, Mark D Rausher

**Affiliations:** ^1^ Department of Biology Duke University Durham North Carolina 27708; ^2^ Department of Ecology and Evolutionary Biology University of Toronto Toronto ON M5S 3B2 Canada; ^3^ College of Horticulture and Landscape Architecture Zhongkai University of Agriculture and Engineering Guangzhou 510225 China

**Keywords:** Gene flow, hybridization, *Ipomoea*, mating system, reproductive barriers, speciation

## Abstract

Hybridization between species can affect the strength of the reproductive barriers that separate those species. Two extensions of this effect are (1) the expectation that asymmetric hybridization or gene flow will have asymmetric effects on reproductive barrier strength and (2) the expectation that local hybridization will affect only local reproductive barrier strength and could therefore alter within‐species compatibility. We tested these hypotheses in a pair of morning glory species that exhibit asymmetric gene flow from highly selfing *Ipomoea lacunosa* into mixed‐mating *Ipomoea cordatotriloba* in regions where they co‐occur. Because of the direction of this gene flow, we predicted that reproductive barrier strength would be more strongly affected in *I. cordatotriloba* than *I. lacunosa*. We also predicted that changes to reproductive barriers in sympatric *I. cordatotriloba* populations would affect compatibility with allopatric populations of that species. We tested these predictions by measuring the strength of a reproductive barrier to seed set across the species’ ranges. Consistent with our first prediction, we found that sympatric and allopatric *I. lacunosa* produce the same number of seeds in crosses with *I. cordatotriloba*, whereas crosses between sympatric *I. cordatotriloba* and *I. lacunosa* are more successful than crosses between allopatric *I. cordatotriloba* and *I. lacunosa*. This difference in compatibility appears to reflect an asymmetric decrease in the strength of the barrier to seed set in sympatric *I. cordatotriloba*, which could be caused by *I. lacunosa* alleles that have introgressed into *I. cordatotriloba*. We further demonstrated that changes to sympatric *I. cordatotriloba* have decreased its ability to produce seeds with allopatric populations of the same species, in line with our second prediction. Thus, in a manner analogous to cascade reinforcement, we suggest that introgression associated with hybridization not only influences between‐species isolation but can also contribute to isolation within a species.

**Impact Statement**

Biological diversity depends on traits that prevent different species from successfully interbreeding. However, these reproductive barriers are often imperfect, leading to hybrid matings and possible genetic exchange between species where they occur together. When this happens, the reproductive barriers that separate species can themselves evolve to become stronger or weaker. Understanding the effects of hybridization on reproductive barriers is key to predicting the potential for future hybridization between species and ultimately whether hybridizing species will diverge, persist, or merge in regions where they co‐occur. Here, we hypothesize and show that hybridization in only one direction causes unidirectional changes to reproductive barrier strength and that geographically restricted hybridization causes local changes in barrier strength that can affect interbreeding within a species. Specifically, we found that gene flow from one species of morning glory into another likely caused a reproductive barrier to decrease in regions where they co‐occur. The decreased reproductive barrier is caused by changes in only the species that received gene flow. We also found that the locally reduced barriers in the species that received gene flow affected reproductive compatibility between populations within that species. Thus, a breakdown of barriers between species can cause a build‐up of barriers within a species. Our work demonstrates critical and rarely explored interactions at species boundaries.

Reproductive barriers are fundamental to the evolution and maintenance of biological diversity. They maintain the integrity of species by preventing hybridization and the homogenizing effect of gene flow. However, reproductive barriers are frequently incomplete, and closely related species often hybridize where they co‐occur (Mallet 2005, Whitney et al. 2010). When hybridization occurs, such as in cases of secondary contact, it often leads to genetic exchange between species and can cause the strength of reproductive barriers to increase or decrease. These changes feedback to affect the potential for subsequent hybridization and gene flow and, therefore, determine whether a pair of species in sympatry will collapse into a single species, complete speciation, or coexist in a stable hybrid zone (Endler 1977, Abbott et al. 2013, Todesco et al. 2016).

Hybridization increases or decreases reproductive isolation in two major ways: (1) by homogenizing genotypes and traits through gene flow and (2) by creating opportunities for selection. First, if hybridization homogenizes genotypes, and thus traits, that underlie reproductive barriers, the strength of reproductive isolation between species can increase or decrease. For example, when a trait that causes assortative mating on its own (e.g., self‐fertilization) spreads to a new species, reproductive isolation will increase (Felsenstein 1981, Ortíz‐Barrientos and Noor 2005). In contrast, homogenization of a trait that produces reproductive isolation through differentiation (e.g., flowering time) will cause isolation to decrease. Second, selection can act to change barriers in ways that depend on the fitness of hybrids. When hybrids are fit, selection may purge alleles that limit potential mates or interspecific fertilizations and thus reduce prezygotic reproductive barrier strength. However, when hybrids are unfit, selection can act either to weed out those unfit hybrids, purging incompatible alleles and reducing postzygotic isolation (Barton and Bengtsson 1986, Gavrilets 1997, Lemmon and Kirkpatrick 2006), or to directly favor earlier‐acting reproductive barriers that limit resources wasted on unfit offspring in a process called reinforcement (Dobzhansky 1940, Blair 1955, Howard 1993, Servedio and Noor 2003).

Which of these trajectories occurs depends on many factors, including the strength and genetic architecture of the initial reproductive barriers, the strength and type of natural selection, and the genetic variation available to selection (Clarke 1966, Felsenstein 1981, Barton and Hewitt 1985, Sanderson 1989, Marshall et al. 2002, Lemmon and Kirkpatrick 2006, Bank et al. 2012, Gompert et al. 2012, Lindtke and Buerkle 2015, Harrison and Larson 2016, Costa et al. 2020). Because these factors are often unknown and are challenging to quantify, it is difficult to predict whether reproductive isolation will increase or decrease in any particular contact zone. However, in cases of asymmetric or geographically restricted hybridization, we can make predictions about how reproductive isolation will change regardless of whether it increases or decreases. Specifically, we expect that asymmetric hybridization will have asymmetric effects on reproductive barriers and that any local change to reproductive barriers due to geographically restricted hybridization may cause reproductive barriers to arise within species.

Asymmetric hybridization and gene flow occur in many organisms (Tiffin et al. 2001, Lowry et al. 2008, Todesco et al. 2016, Abbott 2017). These asymmetries arise under many circumstances, including when one of the two hybridizing species is more common in regions of sympatry (Burgess et al. 2005), is more successful at backcrossing with hybrid offspring (Ippolito et al. 2004), or has a greater genetic load (Bierne et al. 2002, Kim et al. 2018, Pickup et al. 2019). In systems with asymmetric hybridization and gene flow, we expect that any changes to reproductive barrier strength will also be asymmetric. This has been documented in cases of reinforcement (e.g., Noor 1995, Jaenike et al. 2006, Yukilevich 2012), but we also expect it to be the case when barrier strength changes under other circumstances. In the most extreme cases with unidirectional hybridization or gene flow (and no cost of gamete export), we expect that only the species receiving gametes and/or gene flow will experience homogenization, selection against incompatible alleles, or reinforcing selection, and will thus have the potential to evolve in ways that change the strength of isolation.

When species that hybridize do not have completely overlapping ranges, changes to barrier strength in sympatric populations can create reproductive isolation between sympatric and allopatric populations of the same species. This phenomenon has been seen in some cases of reinforcement, where new or strengthened reproductive barriers in sympatry cause incompatibility between sympatric and allopatric populations of the species experiencing reinforcement (e.g., Hoskin et al. 2005, Jaenike et al. 2006, Kozak et al. 2015). This is known as “cascade reinforcement” or “cascade speciation” (Ortíz‐Barrientos et al. 2009) but, much like asymmetry, we do not expect this phenomenon to be limited to reinforcement. Any local change in reproductive barriers is likely to have cascading effects on reproductive isolation within species. For example, if gene flow homogenizes traits causing reproductive isolation through differentiation (e.g., flowering time divergence) in only sympatric populations of a species, sympatric and allopatric populations will become phenotypically mismatched and thus isolated. Similarly, if incompatibility alleles introgress from one species into some populations of another, those alleles will cause incompatibilities between populations that experience introgression and those that do not. Therefore, we expect that both local increases and local decreases in barrier strength can cause barriers to arise within species.

These two hypotheses have seldom been explicitly tested. We test both using a pair of morning glory species that are ideally suited to address these questions. *Ipomoea cordatotriloba* and *I. lacunosa* are sister species that have partially overlapping ranges and exhibit strongly asymmetric introgression from *I. lacunosa* into *I. cordatotriloba* in the regions where they co‐occur (Rifkin et al. 2019a; see below for details). Furthermore, these species are strongly but not completely reproductively isolated by a barrier to seed set that causes interspecific crosses to produce few or no seeds (Martin 1970, Abel and Austin 1981, Diaz et al. 1996, Duncan and Rausher 2013b). We do not know why the crosses fail to set seed, as one or more incompatibilities could manifest anywhere from pollen‐stigma interactions to seed development. Regardless of the mechanism, the strength of the crossing barrier could evolve as a result of homogenization or selection. Although vigorous, hybrids between *I. cordatotriloba* and *I. lacunosa* tend to produce less pollen than the parental species (Abel and Austin 1981; Rifkin 2017). If the hybrids are less fit than the parents (e.g., as a result of reduced pollen production), reinforcing selection could act to increase the crossing barrier, as it acts before maternal provisioning is complete (Coyne 1974, Kay and Schemske 2008, Hopkins 2013). Alternatively, selection could act to lessen the hybrid incompatibility and crossing barrier.

Here, we assess seed set after crosses among sympatric and allopatric populations of *I. lacunosa* and *I. cordatotriloba* to determine whether species co‐occurrence affects the strength of the crossing barrier. Given highly asymmetric introgression from *I. lacunosa* into *I. cordatotriloba* in only the populations where they co‐occur, we have the following two expectations: (1) we expect that, if the crossing barrier evolves, the change will be greater in sympatric populations of *I. cordatotriloba* than in sympatric populations of *I. lacunosa*, and (2) we expect that any change to the crossing barrier in sympatric populations of *I. cordatotriloba* will cascade to cause a reproductive barrier between sympatric and allopatric populations of *I. cordatotriloba*.

## Methods

### SPECIES INFORMATION

**Figure 1:**
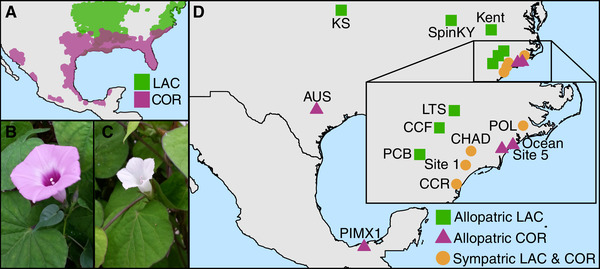
Information about our study species. (A) Map showing the approximate distributions of*I. lacunosa*(LAC, green) and*I. cordatotriloba*(COR, purple) in North America. This map was redrawn based on Khoury et al. (2015) and generally matches collection records from the United States (USDA, NRCS^2019^). (B) A typical allopatric*I. cordatotriloba*flower. (C) A typical*I. lacunosa*flower. (D) Map showing the locations of populations used in this study (see Table S1 for details).

*Ipomoea lacunosa* and *I. cordatotriloba* (Convolvulaceae) are sister species (Muñoz‐Rodríguez et al. 2018) that likely diverged between 1 and 1.6 million years ago (Carruthers et al. 2020). The two species have overlapping ranges in the southeastern United States, likely as a result of recent secondary contact (Rifkin et al. 2019a), but only *I. lacunosa* occurs north of North Carolina into Canada and only *I. cordatotriloba* occurs south and west into more of Mexico (Fig. 1A). Both species produce many bisexual, self‐compatible flowers that open for a single day (Figs. 1B and 1C). However, populations of *I. cordatotriloba* range from nearly complete outcrossing to nearly complete selfing, whereas all populations of *I. lacunosa* are highly selfing (all selfing rates ≥0.89; Duncan and Rausher 2013a). Accordingly, *I. lacunosa* exhibits many traits that are considered part of the “selfing syndrome” (Ornduff 1969, Sicard and Lenhard 2011), including small pale flowers, little nectar, and a low pollen:ovule ratio (Fig. 1C; McDonald et al. 2011; Duncan and Rausher 2013a; Rifkin et al. 2019b).

Rifkin et al. (2019a) identified asymmetric gene flow from highly selfing *I. lacunosa* into mixed‐mating *I. cordatotriloba*. Multiple genetic analyses revealed that *I. lacunosa* is genetically similar across its range, whereas *I. cordatotriloba* consists of two distinct genetic groups that correspond to whether it is allopatric or sympatric with *I. lacunosa* (Rifkin et al. 2019a). Genetic divergence, measured as either π or allele‐frequency differences, between allopatric and sympatric populations of *I. cordatotriloba* is similar to genetic divergence between the species. At the same time, genetic divergence between sympatric *I. cordatotriloba* and *I. lacunosa* is substantially lower than between allopatric populations of the two species. Moreover, sympatric populations of *I. cordatotriloba* contain alleles that are present in *I. lacunosa* but absent from allopatric *I. cordatotriloba*, often at frequencies greater than 0.5. However, the reverse is not common (Rifkin et al. 2019a). These results indicate that there has been substantial introgression from *I. lacunosa* into *I. cordatotriloba* in regions of sympatry in the recent past, whereas there has been essentially no introgression from *I. cordatotriloba* into *I. lacunosa*.

Although introgression from a selfer into a mixed mater is counter to some expectations (Davis and Heywood 1963), a recent survey of gene flow between self‐compatible species with different levels of selfing found more gene flow from selfing species into outcrossing species in every study examined (Pickup et al. 2019). One of several potential explanations for this pattern is that prior self‐fertilization excludes fertilization by interspecific and hybrid pollen in selfing species (Lloyd 1979, Fishman and Wyatt 1999, Goodwillie and Weber 2018, Brys et al. 2016). In these *Ipomoea* species, anthers often dehisce before flowers open, so pre‐emptive self‐fertilization in *I. lacunosa* (where anthers and stigmas touch) is likely to explain at least some of the asymmetry in gene flow.

### POPULATIONS AND CROSSES

To measure variation in the success of crosses made within and between *I. lacunosa* and *I. cordatotriloba* individuals, we grew 60 accessions from 18 populations collected across 14 locations (Fig. 1D; Table S1) under common greenhouse conditions (see Rifkin et al. 2019b for details; these are a subset of the plants used for *Q*_ST_ estimation in that study). Briefly, seeds were scarified and germinated in soil in a growth room under a long‐day cycle (16:8 light:dark) and shifted to a short‐day cycle (12:12 light:dark) after approximately four weeks. When flower buds appeared, the plants were transferred to the Duke University Greenhouse Facility. We selected 40 focal plants made up of 10 individuals from each of the following four population categories: allopatric *I. cordatotriloba*, allopatric *I. lacunosa*, sympatric *I. cordatotriloba*, and sympatric *I. lacunosa* (Table S2). Focal plants were selected to maximize the number of populations represented in each population category and the number of flowers that could be crossed. Allopatric populations were defined as populations growing outside the range of the other species or populations within the range of the other species where only one species was observed after a thorough search. The allopatric and sympatric populations used in this study fall within the genetic groups associated with species co‐occurrence that were identified in Rifkin et al. (2019a).

We reciprocally crossed six flowers from each focal plant and a representative of each population category, so that each focal plant was involved in 48 crosses (two directions × four categories × six crosses), 24 as a pollen recipient and 24 as a pollen donor (Fig. S1). Within each cross type (i.e., all pairwise combinations of the population categories), we made crosses between individuals from at least three populations within each population category, and we did not cross any combination of populations more than three times (Table S2). To perform each of the 1920 crosses, we emasculated flowers the day before they opened by dissecting the corolla and removing the anthers with forceps. Between 0800h and 1200h the next day, we pollinated the emasculated flowers by dabbing anthers from the individual used as the male on the stigma of the flower used as the female. This method transfers many more pollen grains than needed to fertilize the four ovules present in each pistil. In these species, if a flower is left unpollinated or a cross fails, the flower generally abscises and falls off the plant within four days. Therefore, we checked the crosses every day until they abscised or until the seed capsule had dried and the sepals had reflexed, indicating seed maturation. Finally, we counted and weighed the mature seeds and fruits.

### STATISTICAL ANALYSES

To determine whether cross type affected whether a cross was successful, we used R version 3.4.2 (R Core Team 2020) and the R packages *lme4* (Bates et al. 2015) and *glmmTMB* (Brooks et al. 2017) to fit mixed effect models to two measures of cross success: (1) whether a fruit produced at least one mature seed (fruit set; mature seeds were defined as those with seed weight >10 mg; Fig. S2) and (2) the mean number of mature seeds produced by a specific pair of plants (mean seed number). All models included maternal individual nested within maternal population as a random effect. Models of fruit set were fit with binomial error distribution and, because each pair of plants was crossed repeatedly, they included individual cross as a random effect. Models of mean seed number were fit with either a Gaussian or Tweedie error distribution and often included a term for zero‐inflation (see Table S3 for exact model specifications). We also fit models with paternal individual nested within paternal population as a random effect. However, paternal identity was not a significant model term and did not qualitatively change our results (Table S3), and was thus excluded from the results presented below. To determine whether geographic distance affected intraspecific cross success, we fit a model to the mean number of seeds produced (as described above) that also included the distance between the populations of the individuals crosses as a factor. In all cases, we used the R package DHARMa (Hartig 2020) to test model fits for substantial deviations from their expected error distributions (e.g., distribution shape, dispersion, outliers, and zero‐inflation), and we identified significant model terms using likelihood ratio tests and significant contrasts using least‐squared means implemented in the R package *emmeans* (Lenth 2016).

Although all the plants appeared to make healthy and functional flowers, we found that individuals from one allopatric *I. cordatotriloba* population, Ocean, were consistently less fertile than individuals from other populations (only 9% of intraspecific crosses set seed compared to 46‐82% of intraspecific crosses in other populations). We therefore removed this population from all analyses below, but our results do not qualitatively change when this population is included (Table S3).

## Results

**Figure 2:**
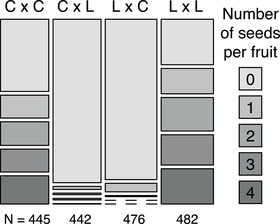
The effect of species combination on seed set. The number of seeds produced by different types of crosses (ovule parent × pollen parent; C =*I. cordatotriloba*and L =*I. lacunosa*). Each box is proportional to the number of fruits that contained indicated number of seeds after pollination. Dashed lines represent cases in which there were no fruits with a particular seed number.

Controlled crosses revealed variation in the success of different cross types. First, our results confirm the existence of a strong reproductive barrier separating *I. cordatotriloba* and *I. lacunosa*. Across all crosses and cross types, 68% of intraspecific crosses and only 5% of interspecific crosses set fruit with at least one mature seed (Fig. 2). Interspecific crosses are significantly less successful than both crosses within *I. cordatotriloba* (Table S3; fruit set: *z* = 14.7, *P* < 0.001; mean seed number: *t* = 4.77, *df* = 295, *P* < 0.001) and crosses within *I. lacunosa* (Table S3; fruit set: *z* = 15.3, *P* < 0.001; mean seed number: *t* = 5.91, *df* = 295, *P* < 0.001). However, we found no evidence that the success of interspecific crosses is affected by which species is used as the maternal parent (Fig. 2; Table S3; fruit set: *z* = −1.12, *P* = 0.262; mean seed number: *t* = 0.84, *df* = 295, *P* = 0.402).

**Figure 3:**
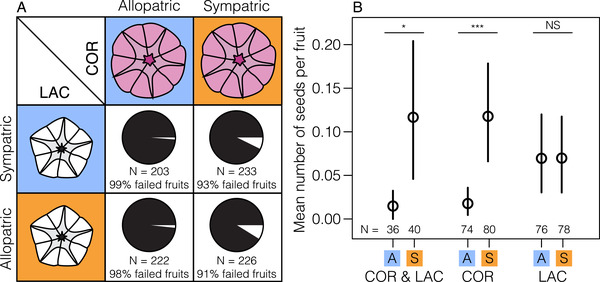
Species co‐occurrence affects interspecific cross success. (A) The proportion of interspecific crosses that set at least one mature seed. (B) The mean and 95% confidence intervals (based on bootstrap percentiles) for the mean number of seeds produced by different types of interspecific crosses (from left to right): both species are allopatric (A), both species are sympatric (S), allopatric*I. cordatotriloba*(COR) and any*I. lacunosa*(LAC), sympatric*I. cordatotriloba*and any*I. lacunosa*, allopatric*I. lacunosa*and any*I. cordatotriloba*, sympatric*I. lacunosa*and any*I. cordatotriloba*.^*^ *P*< 0.05;^***^ *P*< 0.001.

Second, interspecific crosses made with plants from sympatric sites were significantly more likely to be successful than those made with plants from allopatric sites (Fig. 3; fruit set: *z* = −1.98, *P* = 0.048; mean seed number: *t* = −2.03, *df* = 145, *P* = 0.047). To determine whether differences in one or both species explain the pattern of higher seed set in sympatric plants, we compared the success rates of interspecific crosses using sympatric and allopatric plants within each species. We found that crosses between sympatric *I. cordatotriloba* and range‐wide *I. lacunosa* are more likely to succeed than between allopatric *I. cordatotriloba* and range‐wide *I. lacunosa* (Fig. 3; Table S3; fruit set: *z* = −3.11, *P* = 0.002; mean seed number: *t* = −3.09, *df* = 145, *P* = 0.002), whereas crosses between sympatric *I. lacunosa* and range‐wide *I. cordatotriloba* are no more likely to be successful than those between allopatric *I. lacunosa* and range‐wide *I. cordatotriloba* (Fig. 3; Table S3; fruit set: *z* = 0.102, *P* = 0.919; mean seed number: *t* = 0.051 *df* = 145, *P* = 0.959). These comparisons indicate that the higher seed set of interspecific crosses involving sympatric plants is due primarily to higher seed set in crosses involving sympatric *I. cordatotriloba*. Overall, 8% of interspecific crosses that involve sympatric *I. cordatotriloba* are successful, compared to 1.5% of those that do not.

**Figure 4:**
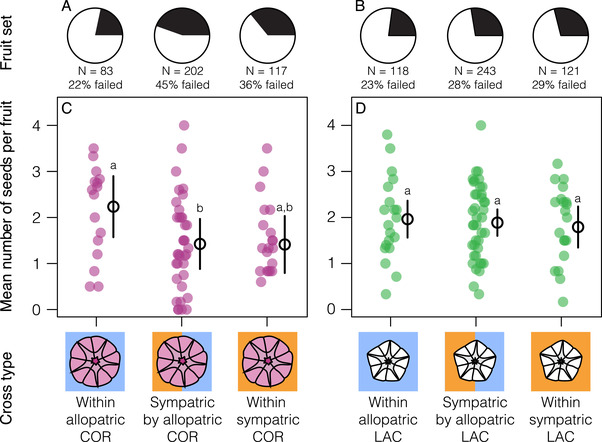
The success of crosses within*I. cordatotriloba*, but not within*I. lacunosa*, depends on species co‐occurrence. The proportion of fruits with at least one mature seed set after crosses within*I. cordatotriloba*(A) and within*I. lacunosa*(B) and the mean number of seeds produced by crosses within*I. cordatotriloba*(C) and within*I. lacunosa*(D). In panels C and D, each point represents the mean number of seeds produced by five to six crosses made with same individuals and means and 95% confidence intervals for those means are also plotted. Means not sharing any letter within a panel are significantly different at*P*< 0.05.

Finally, we found that crosses made within *I. cordatotriloba* were affected by whether the individuals were from allopatric or sympatric populations (Fig. 4A; Table S3; fruit set: $\chi^{2}$ = 13.25, *df* = 2, *P* = 0.001; mean seed number: $\chi^{2}$ = 7.93, *df* = 2, *P* = 0.019), whereas this was not true for crosses within *I. lacunosa* (Fig. 4B; Table S3; fruit set: $\chi^{2}$ = 1.02, *df* = 2, *P* = 0.600; mean seed number: $\chi^{2}$ = 0.38, *df* = 2, *P* = 0.828). Within *I. cordatotriloba*, crosses between two allopatric populations were more successful than crosses between allopatric and sympatric populations (Fig. 4; Table S3; fruit set: *z* = 3.69, *P* < 0.001; mean seed number: *t* = 2.80, *df* = 54, *P* = 0.019) and were marginally more successful than crosses between two sympatric populations (Fig. 4; Table S3; fruit set: *z* = 2.84, *P* = 0.013; mean seed number: *t* = 2.13, *df* = 49, *P* = 0.094). The variation in cross success within *I. cordatotriloba* does not appear to be caused by population variation correlated with geographic distance because geographic distance is not significantly correlated with intraspecific cross success in either *I. cordatotriloba* (Fig. S3; fruit set: $\chi^{2}$ = 1.86, *df* = 1, *P* = 0.173; mean seed number: $\chi^{2}\;$= 2.71, *df* = 1, *P* = 0.099) or *I. lacunosa* (Fig S3; fruit set: $\chi^{2}$ = 0.28, *df* = 1, *P* = 0.596; mean seed number: $\chi^{2}$ = 0.05, *df* = 1, *P* = 0.832). In fact, there is a weak trend in the direction opposite to our expectation, where *I. cordatotriloba* individuals from more distant populations tend to be more compatible (Fig. S3).

## Discussion

We have shown that *I. cordatotriloba* and *I. lacunosa* are separated by a barrier to seed set that is weaker in regions where the two species occur together. This result could explain the wide variation in cross‐compatibility reported in previous studies of these species (Martin 1970, Abel and Austin 1981, Diaz et al. 1996, Duncan and Rausher 2013b). The weaker barrier appears to be caused entirely by changes in the sympatric populations of *I. cordatotriloba*, a result consistent with our hypothesis that asymmetric hybridization and gene flow will lead to asymmetric change in barrier strength. Further, the weaker barrier in sympatric *I. cordatotriloba* likely explains a crossing barrier that we observe between sympatric and allopatric individuals of *I. cordatotriloba*. This result is consistent with our second hypothesis that local decreases in reproductive barrier strength can cause a barrier to arise within a species. Together, our results highlight that, like reinforcement, the erosion of reproductive barriers can be affected by asymmetry and have cascading effects on reproductive isolation. Below we discuss the erosion of reproductive barriers, evaluate whether our results are explained by asymmetric introgression, and consider how the redistribution of barrier alleles might affect species diversification in a manner similar to cascade reinforcement.

Although the basic requirements for reinforcement appear to be met by this system, reinforcement did not occur. This has been observed in other systems (e.g., Ritchie et al. 1989, Coyne et al. 2002, Urbanelli et al. 2014) and could be for many reasons. For example, there could be little selection against the hybrids, gene flow could overwhelm reinforcing selection, or these populations may not harbor the genetic variation needed to respond to selection. Also, in cases of one‐way migration, indirect selection favoring barrier alleles is often too weak to overcome the loss of those alleles through migration (Servedio and Kirkpatrick 1997). Instead of reinforcement, the association between species co‐occurrence and a weaker reproductive barrier suggests that hybridization and gene flow have partially eroded the strength of the crossing barrier. This is noteworthy because, although the erosion of barriers in sympatry is supported by theory (Barton and Hewitt 1985, Butlin 1987, Kelly and Noor 1996) and is implied by patterns of genetic variation in many systems (e.g., Bettles et al. 2005, Borge et al. 2005, Sanders et al. 2014), outside of environmentally induced change (e.g., Mecham 1960, Seehausen et al. 1997, Taylor et al. 2006), studies in which the strength of reproductive barriers has been explicitly shown to be weaker in sympatry are uncommon (but see Virdee and Hewitt 1994, Saetre et al. 1999, Collins and Rawlins 2013). Although this study does not allow us to determine why reinforcement did not occur, it offers insights into the causes and consequences of the erosion of reproductive isolation and allows us to compare the case where barriers weaken to the expectations set by strengthened barriers.

The weaker crossing barrier is likely explained by the homogenization of *I. cordatotriloba* and *I. lacunosa* in sympatry. Consistent with this possibility, hybrids produced by controlled crosses between the species are more cross‐compatible with the parental types than the parents are with each other (see Results in the Supporting Information). Further, the asymmetric erosion of the crossing barrier is consistent with asymmetric introgression of alleles that underlie the barrier from *I. lacunosa* into *I. cordatotriloba*. For example, consider a scenario in which the crossing barrier is caused by incompatible alleles in zygotes (such that zygotes are unlikely to develop into seeds) at a locus that has allele *A* in allopatric *I. cordatotriloba* populations and allele *a* in allopatric *I. lacunosa* populations. When both gametes in a cross carry the same allele, there is no incompatibility, whereas if one gamete is *A* and the other is *a*, there is incompatibility. When secondary contact first occurs, within‐species matings are compatible and between‐species matings are incompatible. After asymmetric introgression, however, the frequency of *a* in the sympatric *I. cordatotriloba* population is high. Therefore, a large proportion of the crosses between this population and sympatric *I. lacunosa* will involve both gametes carrying allele *a*, which are compatible. The crossing barrier between sympatric *I. cordatotriloba* and *I. lacunosa* will thus be lower than between allopatric populations of the two species. We note that this argument is easily extended to multiple loci that interact to cause a breakdown in cross success (see Discussion in the Supporting Information), which is a common form of hybrid incompatibility (Coyne and Orr 2004, Guerrero et al. 2017, Ono et al. 2017).

Our finding that a crossing barrier partially isolates allopatric and sympatric populations within *I. cordatotriloba*, but not within *I. lacunosa*, is also consistent with the asymmetric introgression of alleles that underlie the barrier from *I. lacunosa* into *I. cordatotriloba*. After introgression in the scenario described above, sympatric *I. cordatotriloba* populations would harbor *a* alleles from *I. lacunosa* and would be incompatible with allopatric populations of *I. cordatotriloba* that have *A* alleles. In contrast, sympatric I*. lacunosa* populations, not having received introgression from sympatric *I. cordatotriloba*, would still be fixed for *a* alleles and would not be incompatible with their allopatric counterparts. At the same time, we found some evidence that crosses between sympatric *I. cordatotriloba* populations are less successful than crosses between allopatric populations. This trend can be explained by asymmetric introgression in one of two ways. First, if the introgression of *a* alleles into sympatric *I. cordatotriloba* populations was not complete, *A* and *a* alleles would be segregating in the sympatric populations and could cause crosses made between two sympatric *I. cordatotriloba* individuals to fail. Second, if the crossing barrier has a polygenic basis, incompatibility alleles at different loci could introgress into different sympatric populations, leading to incompatibility between those populations (see Lemmon 2009 for an analogous situation involving reinforcement). Both incomplete introgression and a polygenic basis of the crossing barrier may also explain why the interspecific barrier is only partly eroded in sympatry.

Although the above explanation for the asymmetric change we observe is compelling, it is possible that processes other than introgression explain the asymmetric patterns. First, it could be that *I. cordatotriloba* simply has greater population variation in reproductive barrier strength that happens to correlate with sympatric regions. However, this seems unlikely given that geographic distance does not explain cross success. Second, it could be that a lack of genetic diversity present in *I. lacunosa* (Rifkin et al. 2019a) prevented the evolution of reproductive barriers in the selfing species. In a scenario where there is direct selection on reproductive isolation in sympatry, it is possible that only *I. cordatotriloba* harbored the genetic variation needed to respond. This explanation is more commonly applied to systems that show a lack of reinforcement, but it is theoretically applicable to cases where selection favors weaker barriers as well. Future studies that determine the precise nature and genetic basis of the crossing barrier will allow us to continue to evaluate the merit of these alternative explanations.

It is possible that the asymmetric erosion of the crossing barrier seen here could cause a positive feedback in which that erosion facilitates even more asymmetric gene flow that eventually leads to the extinction of *I. cordatotriloba*. However, it is important to remember that we are only tracking the effect of hybridization on the failure to set seed. Other reproductive barriers (e.g., flowering time and pollinator isolation) could be increasing or decreasing. Indeed, it seems that reproductive isolation caused by differences in the rate of self‐fertilization may have increased in sympatry. Rifkin et al. (2019a) observed less separation between anthers and stigmas and substantially higher selfing rates in sympatric *I. cordatotriloba*. This increased selfing presumably constitutes a barrier to gene flow from *I. lacunosa* (Hu 2015), but this needs to be confirmed by additional experiments. If true, however, the decreased reproductive isolation caused by decreased cross incompatibility we document here may be offset, at least to some extent, by an increase in isolation caused by the effects of gene flow on selfing rate. Interestingly, this may also explain why the crossing barrier was eroded and not reinforced. Simulations by Castillo et al. (2016) showed that increases in self‐compatibility in sympatry often preclude the evolution of other assortative mating traits (e.g., a stronger crossing barrier). Together, the intraspecific crossing barrier and increased selfing in regions of sympatry also suggest that the allopatric and sympatric populations of *I. cordatotriloba* are substantially reproductively isolated.

Our results suggest that the introgression responsible for reduced isolation between the two species in sympatry also contributed to the evolution of increased reproductive isolation between allopatric and sympatric populations of the two species. This process resembles the process of cascade reinforcement, whereby the evolution of increased reproductive isolation between allopatric and sympatric populations of the same species is caused by divergence in sympatry due to reinforcement (Ortíz‐Barrientos et al. 2009, Pfennig 2016). The primary similarity is that gene flow in sympatry results ultimately in evolutionary changes that increase intraspecific isolation. However, unlike reinforcement, where increasing isolation in sympatry contributes to increased isolation within a species, in our system decreasing isolation in sympatry contributes to increased intraspecific isolation. Moreover, although the alleles favored by reinforcing selection do not necessarily originate from introgression, here it is likely that introgressed alleles directly cause the increase in incompatibility between allopatric and sympatric populations of *I. cordatotriloba*. Introgression of alleles that underlie reproductive barriers can have important consequences for species (Zuellig and Sweigart 2018) including by facilitating species diversification (e.g., Pardo‐Diaz et al. 2012, Meier et al. 2017, Todesco et al. 2020). In this case, the redistribution of barrier alleles from between the species to between sympatric and allopatric populations of *I. cordatotriloba* has caused substantial reproductive isolation. This likely explains the extent of genetic divergence observed between these groups in Rifkin et al. (2019a) and suggests that introgression has initiated speciation within *I. cordatotriloba*.

Our study highlights the diversity of outcomes that are possible when hybridization occurs between two species. We found a weaker reproductive barrier in regions where morning glory species co‐occur, and we suggest that introgression is responsible for this evolutionary change. We demonstrate that two phenomena previously considered only in the context of reinforcement (asymmetric and cascading change) also apply to decreases in reproductive isolation. This suggests that introgression can redistribute the alleles that underlie reproductive barriers, even shifting reproductive isolation from between species to between populations within a species and initiating speciation. Future studies that explore associations between species co‐occurrence and reproductive barrier strength should consider the full range of potential consequences of hybridization and gene flow.

## AUTHOR CONTRIBUTIONS

KLO, JLR, and MDR designed the study. KLO and HX collected the data. KLO analyzed the data. KLO, JLR, and MDR drafted the manuscript. All authors read, edited, and approved the final manuscript.

## DATA ARCHIVING

The data and scripts used in these analyses will be made available on an online data repository (Dryad https://doi.org/10.5061/dryad.2bvq83bnx) upon manuscript acceptance.

Associate Editor: Z. Gompert

## Supporting information


**Figure S1**. Diagram of the full crossing design used in this study.
**Figure S2**. Distribution of seed weights produced by crosses in this study.
**Figure S3**. Cross type affects seed number.
**Figure S4**. Cross success is not affected by geographic distance.
**Table S1**. Information about the accessions and populations used in this study. See excel sheet:
**Table_S1**_individuals.xlsx
**Table S2**. List of accessions and populations crosses in this study.
**Table_S2**_focal_groups.xlsx
**Table S3**. Statistical output from all models tested. See excel sheet: Table_S3_statistical_output.xlsx
**Figure S5**. Between species crosses are less successful than any other cross type (COR = *I. cordatotriloba*, BC = backcross, LAC = *I. lacunosa*).
**Table S4**. Statistical output from models of the effects of cross type on fruit set and seed number (BS = between species, CC = within *I. cordatotriloba*, BCC = backcrosses to *I. cordatotriloba*, WF1 = within F1s, BCL = backcrosses to *I. lacunosa*, LL = within *I. lacunosa*).

Supplementary Material

Supplementary Material

Supplementary Material
