# Supplementary material for: Morning glory species co‐occurrence is associated with asymmetrically decreased and cascading reproductive isolation
Source: Evol Lett. 2020 Nov 18;5(1):75–85. doi: 10.1002/evl3.205 (PMC7857285; doi:10.1002/evl3.205)
Supplement: Supplementary file 1 — Figure S1. Diagram of the full crossing design used in this study. Figure S2. Distribution of seed weights produced by crosses in this study. Figure S3. Cross type affects seed number. Figure S4. Cross success is not affected by geographic distance. Table S1. Information about the accessions and populations used in this study. See excel sheet: Table_S1_individuals.xlsx Table S2. List of accessions and populations crosses in this study. Table_S2_focal_groups.xlsx Table S3. Statistical output from all models tested. See excel sheet: Table_S3_statistical_output.xlsx Figure S5. Between species crosses are less successful than any other cross type (COR = I. cordatotriloba, BC = backcross, LAC = I. lacunosa). Table S4. Statistical output from models of the effects of cross type on fruit set and seed number (BS = between species, CC = within I. cordatotriloba, BCC = backcrosses to I. cordatotriloba, WF1 = within F1s, BCL = backcrosses to I. lacunosa, LL = within I. lacunosa). [file EVL3-5-75-s001.pdf]

Supplementary Material for:

Morning glory species co-occurrence is associated with asymmetrically decreased and cascading reproductive isolation

Kate L Ostevik, Joanna L Rifkin, Hanhan Xia, and Mark D Rausher

Correspondence to: kate.ostevik@gmail.com

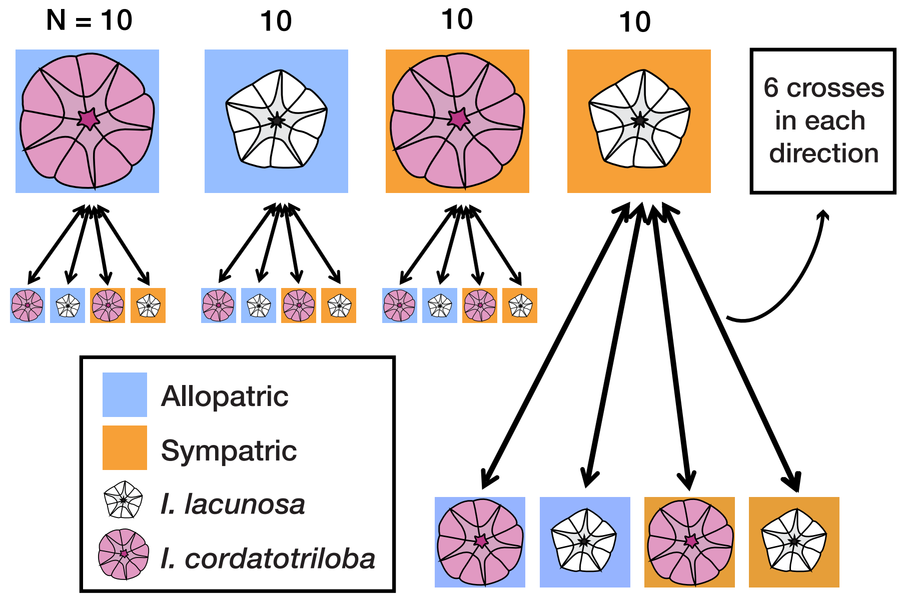

Figure S1 – Diagram of the full crossing design used in this study.

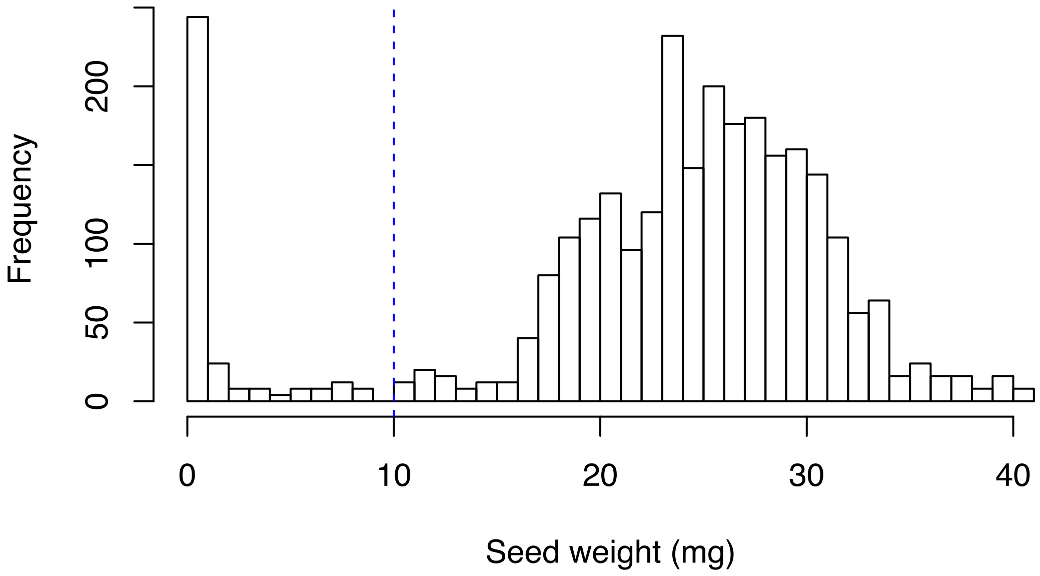

Figure S2 – Distribution of seed weights produced by crosses in this study. We considered seeds that weighed more than 10 mg (dotted blue line) to be mature. Anecdotally, we observed that seeds less than 10 mg appeared lighter in color and more shrivelled and did not germinate, which suggests that they are not fully developed.

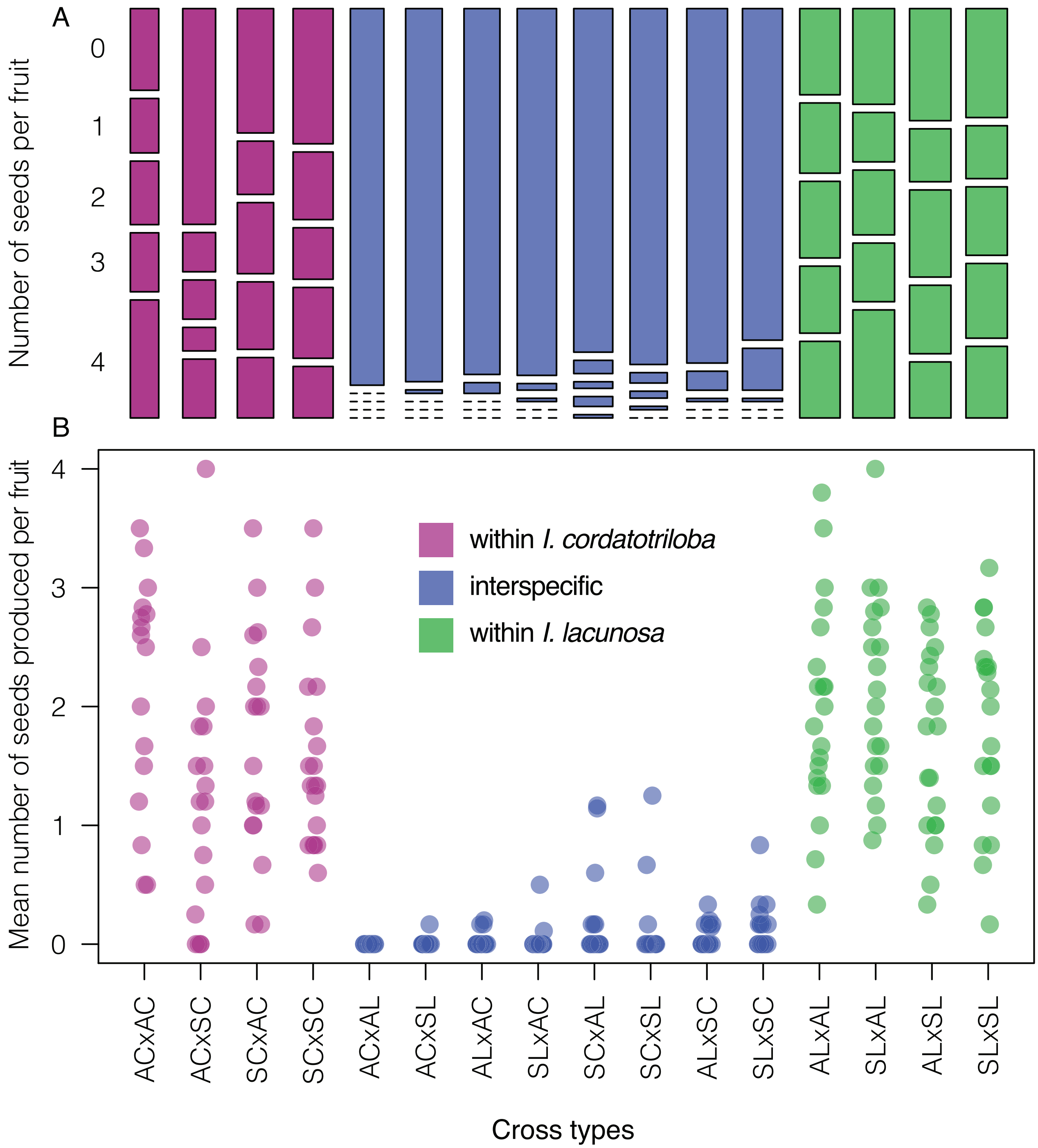

Figure S3 – Cross type affects seed number. A) The number of seeds produced by crosses for all pairwise combinations of population categories: allopatric *I. cordatotriloba* (AC), allopatric *I. lacunosa* (AL), sympatric *I. cordatotrilo*ba (SC), and sympatric *I. lacunosa* (SL). Each box is proportional to the number of fruits that contained the indicated number of seeds after pollination. Dashed lines represent cases in which there were no fruits with a particular seed number. B) The mean number of seeds produced by specific pair of individuals (crosses 5-6 times) grouped based on their population combination.

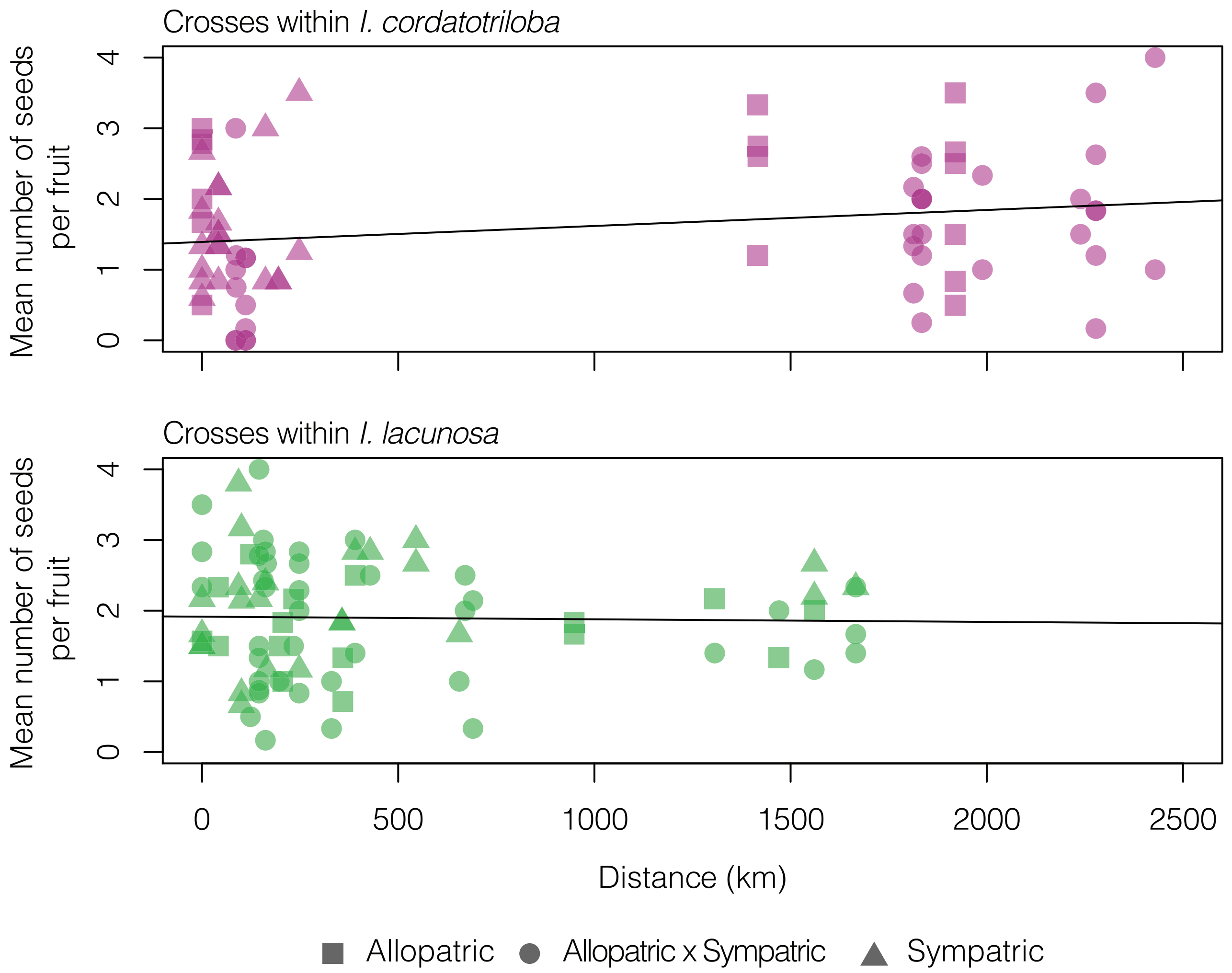

Figure S4 – Cross success is not affected by geographic distance. Here we show the mean number of seeds produced by crosses within *I. cordatotriloba* and *I. lacunosa* versus the geographic distance between where the individuals were collected. Each point represents 5-6 crosses, and shape represents whether the individuals crossed were both from allopatric populations, both from sympatric populations, or from one of each population type. Distance does not significantly affect cross success in *I. cordatotriloba* (distance: = 2.61, df = 1, p = 0.10; note that while not significant, the trend is toward *increasing* compatibility with greater distance) or *I. lacunosa* (distance: = 0.038, df = 1, p = 0.85).

Table S1 – Information about the accessions and populations used in this study. See excel sheet: Table_S1_individuals.xlsx

Table S2– List of accessions and populations crosses in this study. See Table S1 for additional information about each individual (ID) and population (Pop). See excel sheet: Table_S2_focal_groups.xlsx

Table S3– Statistical output from all models tested. See excel sheet: Table_S3_statistical_output.xlsx

Supplementary Methods and Results – Hybrid Crosses

Methods

In a preliminary experiment, we tested the extent to which an inbred line of *Ipomoea lacunosa*, an inbred line of *I. cordatotriloba*, and their F1 hybrids are cross-compatible (inbred lines were derived from wild collected individuals that were collected from the following locations and selfed in the lab for four or five generations: *I. lacunosa* – strain LPR from Kinston, North Carolina [lat: 35.23971, long: -77.57392]; *I. cordatotriloba* - strain CAA from Conway, South Carolina [lat: 33.94713, long: -79.01940]). We grew and crossed pairs of individuals chosen from 38 LAC, 45 COR, and 15 F1 replicates from these inbred lines. To do this, we scarified and germinated seeds on petri dishes in the dark and transferred germinated seeds to soil in a growth room under a long-day cycle (16:8 light:dark). After four weeks, we shifted the growth room to a short-day cycle (12:12 light:dark), and once the plants started to flower, we made controlled crosses using the methods described in the main text. To determine whether cross type affected cross success, we fit linear mixed effect models to fruit set and seed number as described in the main text. Models included maternal and paternal parent as random effects and either a binomial (fruit set) or poisson (seed number) error distribution.

Results

We find that cross type has a significant effect on cross success (fruit set: χ^2 = 73, df = 5, p < 0.001; Seed number: χ^2 = 116, df = 5, p < 0.001) and that crosses made between the parental lines were less successful than any of the other cross combinations (Fig S5; Table S4). This preliminary experiment suggests that F1 hybrid and backcross compatibility is considerably higher than between-species compatibility and indistinguishable from within-species compatibility.

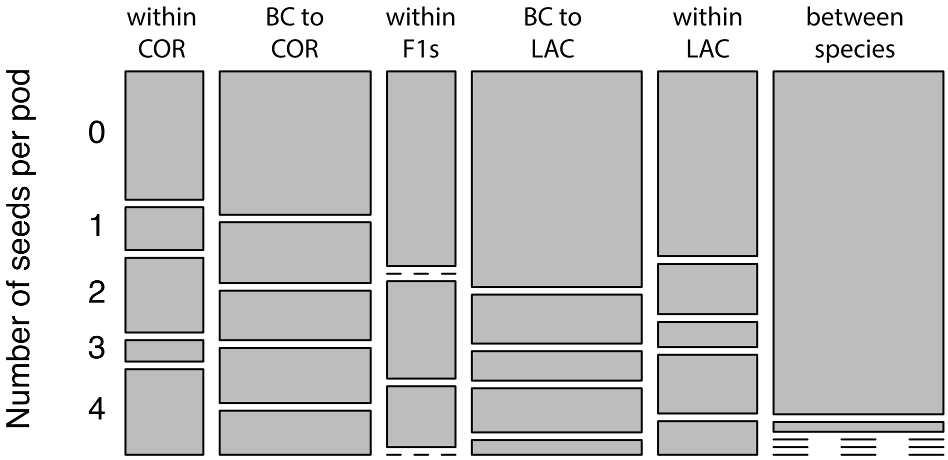

Figure S5 – Between species crosses are less successful than any other cross type (COR = *I. cordatotriloba*, BC = backcross, LAC = *I. lacunosa*). Each box is proportional to the number of fruits that contained the indicated number of seeds after pollination. Dashed lines represent cases in which there were no fruits with a particular seed number.

Table S4 – Statical output from models of the effects of cross type on fruit set and seed number (BS = between species, CC = within *I. cordatotriloba*, BCC = backcrosses to *I. cordatotriloba*, WF1 = within F1s, BCL = backcrosses to *I. lacunosa*, LL = within *I. lacunosa*).

	**Fruit Set**				**Seed Number**			
**contrast**	**estimate**	**SE**	**z.ratio**	**p.value**	**estimate**	**SE**	**z.ratio**	**p.value**
BS – CC	-4.5387	0.926	4.903	<0.0001	-4.1983	0.745	5.636	<0.0001
BS – BCC	-4.4010	0.846	5.202	<0.0001	-4.0081	0.734	5.458	<0.0001
BS – WF1	-3.7331	0.920	4.058	0.0007	-3.8069	0.784	4.853	<0.0001
BS – BCL	-3.4195	0.815	4.195	0.0004	-3.4753	0.742	4.685	<0.0001
BS – LL	-3.7998	0.842	4.511	0.0001	-3.7307	0.743	5.024	<0.0001
CC – BCC	0.1377	0.556	0.248	0.9999	0.1903	0.262	0.727	0.9786
CC – WF1	0.8055	0.730	1.103	0.8802	0.3914	0.41	0.955	0.9318
CC – BCL	1.1191	0.641	1.747	0.5004	0.7231	0.343	2.105	0.2844
CC – LL	0.7389	0.677	1.092	0.8849	0.4676	0.366	1.277	0.7975
BCC – WF1	0.6678	0.563	1.186	0.8438	0.2012	0.290	0.694	0.9827
BCC - BCL	0.9814	0.453	2.167	0.2533	0.5328	0.231	2.306	0.1914
BCC – LL	0.6012	0.542	1.109	0.8778	0.2774	0.307	0.904	0.9457
WF1 – BCL	0.3136	0.538	0.583	0.9922	0.3316	0.284	1.166	0.8531
WF1 – LL	-0.0667	0.644	-0.104	1.0000	0.0762	0.384	0.198	1.0000
BCL - LL	-0.3802	0.482	-0.789	0.9694	-0.2554	0.256	-0.997	0.9192

Supplementary Discussion – Two-Locus Incompatibility Model

We argue here that the observed patterns of incompatibility in this system can be accounted for by a two-locus incompatibility model. Suppose allopatric *I. cordatotriloba* are genotype aaBB at two loci, while allopatric *I. lacunosa* are genotype AAbb, and that crosses with one gamete carrying allele a and one gamete carrying allele b are incompatible. Upon secondary contact and introgression, alleles A and b will be at high frequency in sympatric *I. cordatotriloba* populations. This means that Ab gametes will occur at reasonably high frequencies. Thus, in crosses between sympatric *I. cordatotriloba* and sympatric *I. lacunosa*, both gametes will be Ab and thus compatible, which means that average incompatibility will have been reduced, as is seen in our results. Also, many gametes from sympatric *I. cordatotriloba* individuals will be Ab, which is incompatible with the aB gametes produced by allopatric *I. cordatotriloba* individuals, yielding an average incompatibility between allopatric and sympatric *I. cordatotriloba* greater than zero and accounting for the incompatibility observed between sympatric and allopatric *I. cordatotriloba*.

This model can be extended easily to a situation in which multiple loci each contribute incrementally to incompatibility. In addition, extension to a situation in which incompatibility is produced by interactions between loci that underlie an earlier failure of the cross (e.g., loci that cause a mismatch between pollen and pistils or pollen and ovules) is straightforward.
